# Delayed predictive inference integration with and revision by low-competitive inference alternatives in Chinese narrative text reading

**DOI:** 10.3389/fpsyg.2024.1403479

**Published:** 2024-10-04

**Authors:** Fei Xu, Lulu Cheng, Xianghong Gong, Chenglong Liu

**Affiliations:** ^1^Qingdao University of Technology, Qingdao, China; ^2^School of Foreign Studies, China University of Petroleum (East China), Qingdao, Shandong, China

**Keywords:** predictive inference revision, low-competitive alternatives, integration, activation levels, Chinese narratives

## Abstract

When readers encounter information conflicting with the predictive inferences made earlier, they may update the outdated ones with new ones, a process known as predictive inference revision. The current study examined the revision of disconfirmed predictive inferences by the primarily weakly activated, thus low-competitive inference alternatives during Chinese narrative text reading among Chinese native speakers. We conducted an event-related brain potential (ERP) experiment to study the predictive inference revision with increasingly supportive information for the low-competitive predictive inference alternatives. It serves as the very first attempts to study the predictive inference revision mechanisms by combining a larger range of ERP components, including frontal-Post-N400-Positivity (f-PNP) as an index of revision to examine the influences of the alternative inferences at later stages of reading comprehension. Our results showed that readers could detect inconsistent information (P300), disconfirm the incorrect predictive inferences before successfully integrating the low-competitive alternative predictive inferences with their current situation model (N400), engaging themselves in a second-pass reanalysis process incurring processing costs (P600), and revising the disconfirmed predictive inferences (f-PNP) at a later stage of reading comprehension. Results of this study are supportive of relevant theories in assuming that predictive inference revision does not happen immediately upon encountering conflicting information but happens slowly and incrementally. Our results also unfold the post-revision mechanisms by suggesting the remaining activation and lingering influences of the disconfirmed inferences in the forthcoming reading process.

## 1 Introduction

The human brain is claimed to be a proactive engine (Clark, 2013), which continuously generates predictions for the relevant future (Bar, 2011, p. 13). In language comprehension, predictive inferences are anticipations about the likely outcomes of a described event, typically featured by indication of what will happen next (Calvo and Castillo, 1996). If comprehenders make predictive inferences that allow them to integrate information more easily, they will build more coherent narrative representations (Klin et al., 1999a). When readers are likely to make explanatory predictions that contradict with the following information, they will significantly slow their reading speed (Klin et al., 1999a; van den Broek et al., 2015). This suggests that building a coherent narrative representation is disrupted when adults experience conflicting information related to explanatory predictive inferences. As a result, the procedure of updating the disconfirmed predictive inferences could have happened from time to time since not all expectancies from readers could perfectly match the follow-up information (Nahatame, 2014; Pérez et al., 2015, 2019; Wright and Newhoff, 2002).

Though predictive inference revision is essential for achieving consistency in the situation model construction, there had been discrepancies in whether the inconsistent predictive inferences could be revised (e.g., Nahatame, 2014; Pérez et al., 2015, 2019; Wright and Newhoff, 2002). Some studies found that readers have difficulties suppressing the contradictory predictive inferences (e.g., Potts et al., 1988; Xu et al., 2023) even though there could be possibilities of disconfirmation and revision of the improper predictive inferences (e.g., Wright and Newhoff, 2002; Iseki, 2006). What’s more, previous studies investigated temporary syntactic ambiguity processing in “garden-path” sentences, which contain a local syntactic or semantic ambiguity that initially leads comprehenders to interpret the sentence in a way that is not consistent with the following input (Ceháková and Chromý, 2023). These studies discovered that syntactic reanalysis happened once comprehenders encountered inputs that were inconsistent with their previous analysis (Frazier and Rayner, 1982). It was believed that prediction revision was not necessary even though reanalysis was necessary for comprehenders to successfully interpret garden-path sentences. Although comprehenders did not revise their predictions when encountering prediction-inconsistent cues, they were still capable of processing the relevant input when it arose. Moreover, since prediction revision was a time and cognitive resource-consuming procedure, it was not worthwhile for the language processing system to revise disconfirmed predictions in some cases, because the relevant input might become available immediately after the prediction-inconsistent cue, or even before a revised prediction could be computed (Chow and Chen, 2020).

However, despite all evidence or assumptions disapproving the possibility of successful revision, some studies did detect revision of improper predictive inferences and the procedure is influenced by factors such as readers’ working memory (e.g., Pérez et al., 2015, 2016), cognitive control abilities (e.g., Pérez et al., 2019), or second language proficiency (e.g., Nahatame, 2014; Pérez et al., 2019). These findings indicate the potential of successful revision when initial predictive inferences were contradicted by inconsistent follow-up information. It can also be seen that many factors could exert influences on predictive inference revision. Moreover, among the studies that have discovered successful revision, there have been conflicting views concerning how quickly the predictive inference revision might happen when the initial inferences are disconfirmed by new information. Some studies have demonstrated that comprehenders could process language incrementally by quickly integrating various sources of information and constraints (e.g., Tanenhaus et al., 1995; Kamide et al., 2003), suggesting that comprehenders would revise the disconfirmed predictions immediately after encountering disconfirming evidence in the input. However, according to the assumptions in the Knowledge Revision Components framework (KReC, Kendeou and O’Brien, 2014), the knowledge revision process was conceptualized as incremental, conservative, and slow. Predictive inference revision can be defined as a type of knowledge revision procedure referring to the modification of the existing knowledge base in memory to accommodate newly acquired information (Kendeou and O’Brien, 2014). Therefore, the KReC framework has the potential of explaining the predictive inference revision procedure. Only when the amount and the quality of new information integrated into the knowledge base crosses some threshold could overt evidence of knowledge revision become evident (Kendeou and O’Brien, 2014). Therefore, readers might need an accumulative amount of information to either disconfirm or revise the conflicting predictive inferences at a time later than when the mismatch has been discovered.

Still, the disruptive effects of the out-of-date predictive inferences and their deletion from readers’ working memory have rarely been investigated. In fact, even if readers have disconfirmed the incorrect predictive inferences, chances are that they could hardly replace them with correct ones. Even though predictive inference revision could happen, it is also possible that information disconfirming a strong prediction may have a disruptive effect on processing, thus preventing comprehenders from detecting inconsistent cues with the input or immediately revising their previous predictions (Federmeier et al., 2007; Van Petten and Luka, 2012; Chow and Chen, 2020). As indicated by studies and theoretical frameworks in knowledge revision, the disconfirmed information may still stay in the working memory. This out-of-date information still has chances to be reactivated when new information comes in to support it (Rapp and Kendeou, 2007; Kendeou and O’Brien, 2014). This shed lights on potential difficulties in avoiding possible interferences of the disconfirmed inferences by deleting the incorrect predictive inferences from readers’ working memory.

In addition, comprehenders could generate more than one predictive inference while reading, so there could be predictive inference alternatives besides the primarily generated predictive inference. Yet prior research failed to reach an agreement on what effects alternative predictive inferences have on the generation and encoding of predictive inferences. Some detected failure of predictive inference making as a result of interferences from the inference alternatives (Klin et al., 1999a). Other researchers found that predictive inferences could be activated and encoded into readers’ mental representation despite the presence of alternative predictive inferences (e.g., Cranford and Moss, 2019; Weingartner et al., 2003). The situation for predictive inference revision will be more complex when the primary predictive inferences are to be replaced by their alternatives even when the primary inferences are strongly supported by the contexts. Therefore, further investigations are necessary to find out the revision procedure of inconsistent predictive inferences with more information coming in to support the now-consistent predictive inference alternatives (Xu et al., 2023).

Among the very few studies concerning the predictive inference revision procedure by employing the ERP technique (e.g., Pérez et al., 2015), two subcomponents of P300, namely P3a and P3b are considered to reflect the mechanisms of attentional control when new information appears (see Friedman et al., 2001; Pérez et al., 2015) and the processing capacity of updating the once-activated but no-longer-relevant information in a revision process (Kok, 2001; Pérez et al., 2015), respectively. P3a was used as an index of a top-down, stimulus-driven process taking place in the frontal areas; P3b an index of a bottom-up updating process in the parietal areas (see Polich, 2003). As reviewed by Van Petten and Luka (2012), abundant evidence revealed the close relationships between P3b and disconfirmation of an expectation. Another ERP component closely related to the current study is the N400, which has functionally been interpreted as reflecting ease of semantic integration according to the integration view (e.g., Hagoort et al., 2004), lexical retrieval according to the lexical view (e.g., Kutas and Federmeier, 2011), or both integration and retrieval on more recent “hybrid” accounts (e.g., Nieuwland et al., 2020). For indexes reflecting the predictive inference revision costs, existing literature considers the modulation of the amplitude of the late posterior positivity/P600 effect to be connected with the efforts readers make when plausible language input contradicts the prediction already made (e.g., Federmeier et al., 2007; Federmeier et al., 2010). As suggested by previous studies, a processing cost is incurred when a specific “high-certainty” prediction is disconfirmed (whether that prediction concerns an event, structure, or thematic role assignment), and that this cost is reflected in the P600 (Kuperberg, 2013; Boudewyn, 2015). Different from the posterior late frontal positivity effect, f-PNP is a late, frontally distributed positivity, which is maximal over frontal electrode sites. This late, frontally-distributed positivity is an effect first characterized as a response to plausible but unexpected words appearing in highly constraining sentence contexts (Brothers et al., 2020). The ERP component reflects detection of a lexical prediction violation, inhibition of an incorrectly predicted word (Kutas, 1993; Federmeier et al., 2007), and/or the incorporation of new and unexpected information into a higher-level representation of meaning (Brothers et al., 2020; Kuperberg et al., 2020). Thus, the f-PNP component has been related to comprehenders’ efforts of attempting to update expectations for future reading (Kuperberg and Jaeger, 2016) or of revising a mis-prediction that has come in conflicts with a specific lexical item (Zirnstein et al., 2018).

There are two hypotheses for the current study. On the one hand, readers could succeed in fulfilling the whole sub-processes of predictive inference revision including mismatch detection when new information conflicts with the primary predictive inferences, inconsistent primary inference disconfirmation before successful integration of the alternative predictive inferences and updating of the inconsistent primary predictive inferences. On the other hand, there is delayed integration of the low-competitive alternative predictive inferences and revision of the disconfirmed but primarily strongly activated predictive inferences when more information comes in to support the initially low-competitive predictive inference alternatives. The primarily generated but later disconfirmed predictive inferences still exert influences at later reading comprehension stages after successful detection of conflicts between the inconsistent predictive inferences and new information input.

Generally speaking, the predictive inference revision procedure may include such sub-processes as mismatch detection, inconsistent information disconfirmation, integration of consistent alternative predictive inferences and updating of the inconsistent predictive inferences. In our study, P3a serves as a mismatch detection index, and P3b as the index of disconfirmation of incorrect predictive inferences (see also Xu et al., 2023). We also take N400 as an index reflecting the integration of the alternative predictive inferences. P600 is taken as an index related to costs that are associated with disconfirmed predictions and f-PNP serves as an index of inconsistent predictive inference updating or revision. The current study is among the first ones to investigate the predictive inference revision process when alternative predictive inferences are present after more information comes in to support the alternative predictive inferences instead of an immediate examination of the process upon encountering the conflicting information. The current research derived from the contradiction paradigm and enjoys the advantage of including separate stages of predictive inference making and contradiction of inconsistent predictive inferences with new information. The last sentence in the ERP was the same in both conditions except that they were ended up with two different words representing either the originally generated but later disconfirmed predictive inference (hereafter referred to as Target 1) or the alternative predictive inference (hereafter referred to as Target 2). The research contains two with-group independent variables. The first variable is consistency of information contained in the fourth sentence of each short narratives with the primary generated predictive inferences. The other variable is target word types ending the ERP sentence. Target 1 is in accord with the primary predictive inference while Target 2 is in line with the originally low-competitive predictive inference alternative. The dependent variable of the current research is mean amplitude of different ERP components from the target words. As a result, we could innovatively further the investigation of the revision procedure when more information comes in to support the once-weakly activated alternative inferences. Differences in the electrophysiological activities of the two different ending words would indicate the activation levels and encoding forms of both the primary and alternative predictive inferences after readers have encountered the disconfirming information.

## 2 Method

### 2.1 Participants

Thirty-four Chinese students (13 males and 17 females, *M_age_* = 20.10, range 18–23, *SD* = 1.35) from a university participated in the current study. All participants were native speakers of Mandarin Chinese with no history of neurological disease or psychiatric disorders. All participants were right-handed as assessed by the Edinburgh Handedness Inventory (Oldfield, 1971) (*M* = 0.85, range 0.60–1, *SD* = 13.19) indicating right-handedness. Participants also engaged themselves in a Digit Span Forward task (*M* = 10.37, range 8–12, *SD* = 1.07) with a total score of 12 and a Digit Span Backward task (*M* = 8.07, range 5–10, *SD* = 1.74) with a total score of 10 (Gong, 1983), indicating high levels of working memory for participants.

### 2.2 Materials

A 63 (3 practice and 60 experimental) short passages were chosen from 192 short passages. These short narratives were based on typical daily events. We chose 63 short passages out of the 192 passages by two norming procedures, namely, a cloze procedure for another group of participants to infer what might happen next after reading the first sentences in each passage by writing down two two-character Chinese verbs and a rating procedure by still another group of participants for appraisals in a probability judgment test on a 7-point Likert-type Scale. Details for the norming study of the 63 passages used in the current experiment can be found in Xu et al. (2023). These 63 passages had strong contextual constraints inducing a main strongly activated predictive inference and a weaker alternative through rating. There were five sentences in each passage. Either the contents or the wording of the first four sentences were in exact accordance with those used in Experiment 2 of Xu et al. (2023). The introduction (Sentences 1–3) was kept together with the fourth sentence in the revise condition, which contains conflicting information with the primarily generated predictive inferences and the no revise condition strengthening the possibility of the primary inference as well as a neutral condition continuing merely the story. The first four sentences were followed by an ERP sentence in two versions. The ERP sentence in two versions continued the whole story and differed in the disambiguating words (either Target 1 or Target 2) that ended the ERP sentence. The ERP sentences were modified very carefully to ensure that they could continue the story in a natural way. An example experimental material is shown in Table 1.

**Table 1:** Sample material used in the experiment.

Introduction	Bias 抄袭(Chāo Xí)	小明通过这次考试才能毕业。 他看到最后一个题目就蒙了。 他偷偷地瞥了监考老师一眼。
	English translation bias cheat	English translation Xiaoming could graduate only if he passed this examination. He was puzzled at the sight of the last question. He peeped at the supervisors of the examination.
Revise sentence	他收拾好试卷走向监考老师。 English translation He took up his answer sheet and walked to the supervisors.	
ERP sentence 1	他实在是不会做就打算**抄袭**(Chāo Xí)。 English translation Unable to answer any more questions, he decided to ***cheat***.	
ERP sentence 2	他实在是不会做就打算**交卷**(Jiāo Juàn)。 English translation Unable to answer any more questions, he decided to ***submit*** the answer sheet.	
Comprehension question	他看到最后一个题目蒙了吗? English translation Was he puzzled at the sight of the last question?	

Note: “Introduction” in the table provides a high contextual support to help elicit a primary predictive inference; “Revise sentence refers to the fourth sentence of each story containing conflicting information with the primary predictive inference; “ERP sentence 1” is ended with a word in bold (in Chinese or English) representing the primary predictive inference; “ERP sentence 1” has a word (in Chinese or English) in bold representing the alternative predictive inference at the end; “Comprehension question”.

The 63 five-sentence long Chinese narratives were followed by comprehension questions encouraging participants to have a good comprehension of the contents. The target words consisted of action verbs of two Chinese characters with no significant difference between the word frequencies of Target 1 (*M* = 15.16, *SD* = 22.14) per million and Target 2 (*M* = 14.33, *SD* = 23.24) per million (Cai and Brysbaert, 2010), *t*(59) = −1.20, *p* = 0.23 or between the number of strokes for Target 1 (*M* = 18.13, *SD* = 4.75) and Target 2 (*M* = 17.07, *SD* = 4.34), *t*(59) = 1.34, *p* = 0.18. In order to make sure that all short narrative texts were globally consistent, a rating task was carried out. Thirty-one participants with similar ages and educational background took part in the rating task judging whether the short narratives were globally coherent or not, on a seven-point scale ranging from 1 (extremely coherent) to 7 (extremely incoherent) with 4 being neutral. Results of *t*-tests showed that the 5-sentence short narratives were considered as globally coherent both in Target 1 condition (*M* = 4.67, *SD* = 0.22) and Target 2 condition (*M* = 4.65, *SD* = 0.20), with no significant differences between the two conditions, *t*(59) = 0.66, *p* = 0.51.

### 2.3 Procedure

Stimuli were presented using E-Prime software (Schneider et al., 2002) and were displayed on a 19-inch. CRT video monitor with a refresh rate of 75 HZ on a black background. Each trial began with a cross “+” in the middle of the screen with a duration of 750 ms. The first four sentences in each passage were presented one by one. Participants rested their right thumbs on the line-advance key and pressed the key to erase the present sentence while presenting the next one. The ERP sentence, or the fifth sentence, was presented word by word with a duration of 300 ms. There was also a delay of 700 ms after the disambiguating word to ensure successful recording of the electrophysiological activities for a sufficiently long time-window. Participants were required to try not to blink during the presentation of words in the ERP sentence. Participants were requested to response to a true/false comprehension question after reading the ERP sentence. Participants pressed the designated true (“m”) or false key (“z”) to give a response. Each of the 60 sets of experimental texts (in two conditions) assigned into two versions were counterbanlanced and presented to every participant only once, keeping 10 randomized passages in each condition per block (altogether 6 blocks). Same numbers of participants were engaged in each condition. Participants in the practice section should achieve more than 90% accuracy rate in three trials to make sure that they had comprehended and followed the instructions well. The whole experiment lasted for about 45 min.

### 2.4 EEG recording and analysis

Electroencephalogram (EEG) was recorded using Ag-AgCl electrodes mounted on an elastic cap (NeuroScan Inc., Herndon, VA, USA) from 64 active electrodes in a 10/20 system montage. Signals were sampled at 1000 Hz and filtered offline at 0.1–30 Hz. We collected ocular movements and blinks by two pairs of channels. The first pair was the vertical electrooculogram situated in the left eye of the participant, with one electrode supra and another infraorbitally to measure blink artifact. The other pair was the horizontal electrooculogram placed in the external canthi, with one electrode on the left and another on the right side to register eye movements. Impedances were kept below 5 kΩ. EEG preprocessing and ERP analysis were conducted with Curry 8.0 software. Ocular artifacts were eliminated from the data. Epochs containing other artifacts were rejected (1.67%) with potentials exceeding ±100 μv. ERP epoch length was1000 ms and a 200 ms pre-stimulus baseline correction was applied. Individual averages were re-referenced off-line to the average of left and right mastoids. Time windows of interest for ERP analysis were determined by visual inspection and previous studies. Analysis of P3a, P3b, and N400 followed such ROIs based on Pérez et al. (2020) to show the mismatch detection, alternative predictive inference disconfirmation and integration of the alternatives. The six regions of interest (ROI) were chosen out of the 64 electrodes, each containing five electrodes based on electrode sagitality and laterality. The five sites of electrodes were grouped between the two hemispheres (left and right) as the left frontal (LF, averaging among F1, F3, F5, FC3, and FC5); the right frontal (RF: F2, F4, F6, FC4, and FC6); the central (C: C1, C2, CZ, FCZ, and CPZ); the left parietal (LP: P1, P3, P5, CP3, and CP5); the right parietal (RP: P2, P4, P6, CP4, and CP6), and the occipital (O: O1, O2, POZ, PO3, and PO4). Posterior-PNP or P600 mean amplitude was measured across 11 parietal-occipital channels (CP1, CP2, CZ, CP5, CP6, P3, P4, P7, P8, O1, O2) to capture posterior positivity effects (similar to Kuperberg et al., 2006; Rasenberg et al., 2020). For f-PNP, there have been a mixture of electrode choices (see Zirnstein et al., 2018; Kuperberg et al., 2020; Hodapp and Rabovsky, 2021). We chose six frontal electrodes, i.e., F1, F3, F5, F2, F4, and F6 showing the most prominent f-PNP effect similar to Zirnstein et al. (2018) for predictive inference revision analysis.

## 3 Results

**Figure 1:**
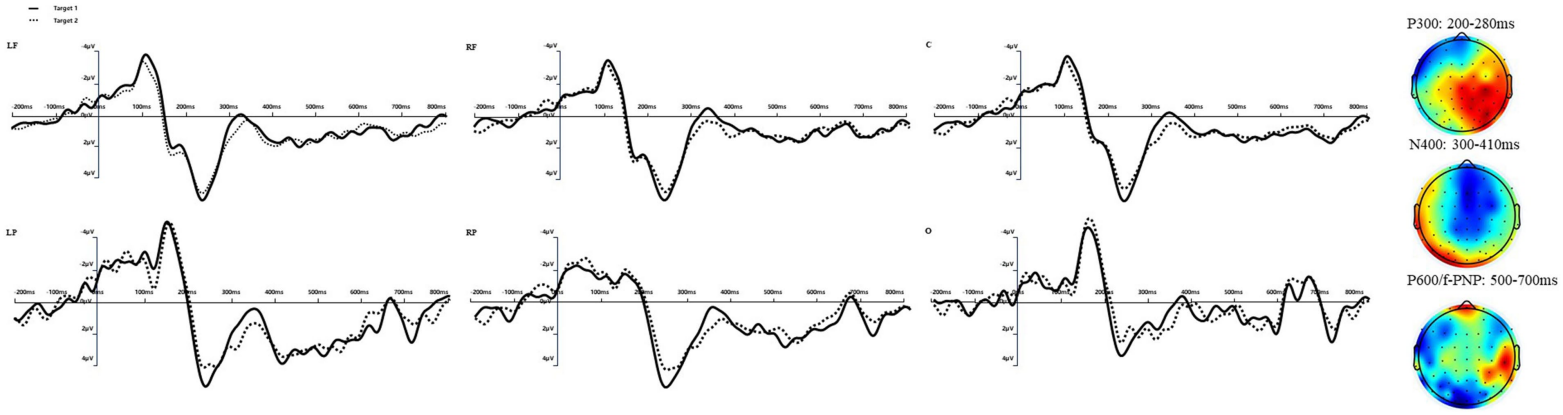
Left: Graphical representation of the mean amplitude (in microvolts) for P300, N400, P600 and f-PNP components divided by contextual conditions and ROIs. Right: Topographic distribution of ERP effects in the 200-280 ms, 300-410 ms, 500-500 ms, and 500-700 ms time windows.

Our analysis of ERP components across various conditions and regions of interest (ROIs) showed significant differences in brain activities, highlighting the intricate nature of cognitive processing involved. Below, we will detail these findings by structuring around key ERP components. Data from four participants were discarded: two due to blinking artifacts and two due to low accuracy rate in answering comprehension questions. Both correct and incorrect responses were included for statistical analysis of 30 participants for all trials. The mean amplitude was calculated in the time window of 200–280 ms for the ERP component of P3a and P3b, the time window of 300–410 ms for the ERP component of N400, 500–700 ms for the ERP components of P600 and f-PNP, after the target word onset. Figure 1 shows the ERPs and difference wave for Target 1 and Target 2 averaged over LF, RF, C, LP, RP, and O electrodes, as well as the voltage maps. We identified N400 at the six ROIs in the time window of 300–410 ms. There was also shorter time windows as in Aurnhammer et al. (2021) reporting the *p*-values for the N400 time window as 350–450 ms. Outlier amplitude data per condition, and ROIs were detected by the Box-Whisker plot and replaced by the mean for P300 (2.03%), N400 (3.11%), P600 (1.2%), and f-PNP (8.89%).

The P3a, a positive ERP component that is maximal at central electrodes and peaks around 300 ms after word onset has its neural sources in regions such as the auditory cortex, parietal lobe, and prefrontal regions (Linden, 2005; Polich, 2007). We conducted a 2 (target word types: Target 1 and Target 2) × 6 (ROIs: LF, RF, C, LP, RP, and O) repeated measures ANOVA on the mean amplitude data to see whether P3a was more significant in the frontal areas and the P3b more significant in the parietal areas. Results showed a significant main effect of contextual conditions, *F*(1, 29) = 13.42, *p* = 0.001, *η^2^_p_* = 0.32. We found more positive amplitude in Target 1 (*M* = 3.63, *SD* = 3.08) than Target 2 (*M* = 2.98, *SD* = 3.11). We also found a main effect of ROIs, *F*(5, 145) = 10.39, *p* < 0.001, *η^2^_p_* = 0.26, the central-frontal regions being more positive than the posterior regions. We carried out a follow-up repeated measures ANOVA on mean amplitudes in different ROIs (central frontal: LF, RF, C, and posterior: LP, RP, and O), *F*(5, 295) = 20.47, *p* < 0.001, *η^2^_p_* = 0.26, and found the central frontal areas being more positive, specifically, LF (*M* = 3.98, *SD* = 2.74), RF (*M* = 4.24, *SD* = 2.99), and C (*M* = 4.50, *SD* = 3.10) than in the posterior areas, specifically LP (*M* = 2.33, *SD* = 3.09), RP (*M* = 3.20, *SD* = 2.60) and O (*M* = 1.57, *SD* = 3.11). We failed to find significant interactive effects between target word types and ROIs, *F*(5, 145) = 1.23, *p* = 0.30, *η^2^_p_* = 0.04. Based on these analyses, we conducted separate analyses of the P3a in the central-frontal areas (LF, RF, and C) and the P3b in the posterior areas (LP, RP, and O).

### 3.1 P3a analysis for mismatch detection

For answering all the questions above, the mean amplitude data was analyzed to see if participants could detect the mismatch between Target 1 from the information disconfirming the primarily generated predictive inferences and the introduction (Sentences 1–3). If participants could detect the mismatch, there should be a reduction in the mean amplitude data in ROIs associated with P3a while reading Target 2 representing the meaning of alternative predictive inferences compared with Target 1representing the meaning of the primary predictive inferences. We carried out a 2 (target word types: Target 1 and Target 2) × 3 (ROIs: LF, RF, and C) repeated measures ANOVA on the mean amplitude data in the time window of 200–280 ms. We found significant main effect of target word types, *F*(1, 29) = 11.15, *p* = 0.002, *η^2^_p_* = 0.28. The mean amplitude for ROIs related with P3a in Target 1 (*M* = 4.59, *SE* = 0.53) were significantly more positive than that in Target 2 (*M* = 3.89, *SE* = 0.51). We did not find significant main effect of ROI, *F*(2, 58) = 2.73, *p* = 0.07, *η^2^_p_* = 0.09. We failed to find significant interaction between the contextual conditions and ROIs, *F*(2, 58) = 2.20, *p* = 0.12, *η^2^_p_* = 0.07, either.

### 3.2 P3b analysis for disconfirmation of inconsistent inferences

According to the hypothesis stated above, readers could further deactivate the revised predictive inference and enhance the activation levels of the alternative predictive inference when supported by more incoming information. We analyzed the mean amplitudes of Target 1 and Target 2 ERPs in the 200–280 ms at posterior and occipital electrodes with a repeated-measures ANOVA. There was a significant main effect of target word types, *F*(1, 29) = 11.54, *p* = 0.002, *η^2^_p_* = 0.29. We found significantly more positivity with Target 1 (*M* = 2.67, *SE* = 2.90) than Target 2 (*M* = 2.07, *SE* = 3.08). We also detected a significant main effect of ROIs, *F*(2, 58) = 10.44, *p* < 0.001, *η^2^_p_* = 0.27. There was significantly more positivity in RP (*M* = 3.20, *SE* = 0.46) than LP (*M* = 2.33, *SE* = 0.56) than O (*M* = 1.57, *SE* = 0.56). We did not find significant interaction between the contextual conditions and ROIs, *F*(2, 58) = 1.69, *p* = 0.19, *η^2^_p_* = 0.06.

### 3.3 N400 analysis for delayed alternative inference integration

According to our hypothesis, readers could integrate the alternative predictive inferences when they read Target 2 at the end of the ERP sentence. We conducted a 2 (target word types: Target 1 and Target 2) × 6 (ROIs: LF, RF, C, LP, RP, and O) repeated measures ANOVA on the mean amplitude data for N400 in the 300–410 ms time window. This analysis showed a significant main effect of target word types, *F*(1, 29) = 5.50, *p* = 0.03, *η^2^_p_* = 0.16, with less negativity for Target 2 (*M* = 1.13, *SE* = 2.29) compared with Target 1 (*M* = 0.60, *SE* = 0.32). We found no significant main effect of ROIs, *F*(5, 145) = 2.30, *p* = 0.11, *η^2^_p_* = 0.07. No significant two-way interaction effect between target word conditions and ROIs was found, *F*(5, 145) = 1.83, *p* = 0.16, *η^2^_p_* = 0.06, either.

### 3.4 P600 analysis for disconfirmation costs

In accordance with the above hypothesis, readers could carry out re-analyses when primarily generated predictive inferences were in conflicts with the new input when reading Target 2 at the end of the ERP sentence. We analyzed the mean amplitudes of target words with a 2 (target word types: Target 1 and Target 2) × 11 (electrodes: CP1, CP2, CPZ, CP5, CP6, P3, P4, P7, P8, O1, and O2) repeated measures ANOVA for P600 component in the time window of 500–700 ms. We found a significant main effect of target word types, *F*(1, 29) = 7.44, *p* = 0.030, *η^2^_p_* = 0.15. There was significantly more positivity tendency with Target 1 (*M* = 0.99, *SE* = 0.18) than Target 2 (*M* = 0.78, *SE* = 0.15). We also found a significant main effect of electrodes, *F*(10, 290) = 10.22, *p* < 0.001, *η^2^_p_* = 0.26, with CP1 (*M* = 1.37, *SE* = 0.23), CP2 (*M* = 1.14, *SE* = 0.21), CPZ (*M* = 1.18, *SE* = 0.22), and CP5 (*M* = 1.22, *SE* = 0.17) generally having larger amplitude than other electrodes. We also found a significant two-way interaction effect between target word types and electrodes, *F*(10, 290) = 18.71, *p* < 0.001, *η^2^_p_* = 0.39. Simple effects analysis showed that there were significant effects for the 11 electrodes in both target word conditions and there were significant effects for target words at electrodes such as CP2, CPZ, P4, and CP6.

### 3.5 f-PNP analysis for delayed inconsistent inference updating

To detect whether readers could revise the disconfirmed predictive inferences when they read Target 2 at the end of the ERP sentence, we conducted a 2 (target word types: Target 1 and Target 2) × 6 (electrodes: F1, F3, F5, F2, F4, and F6) repeated measures ANOVA for f-PNP in the six electrodes in the 500–700 ms. A significant main effect of target word types, *F*(1, 29) = 6.42, *p* = 0.02, *η^2^_p_* = 0.18, with significantly more positivity tendency with Target 1 (*M* = 1.25, *SE* = 0.21) than with Target 2 (*M* = 0.84, *SE* = 0.21) was found. There was a significant main effect of electrodes, *F*(5, 145) = 3.74, *p* = 0.003, *η^2^_p_* = 0.11. The frontal areas including F2 (*M* = 1.23, *SE* = 0.23), F1 (*M* = 1.22, *SE* = 0.21), F3 (*M* = 1.06, *SE* = 0.18), and F4 (*M* = 1.05, *SE* = 0.25) had bigger amplitudes than F5 (*M* = 0.84, *SE* = 0.15) and F6 (*M* = 0.89, *SE* = 0.23). There was no significant two-way interaction effect between contextual conditions and electrodes, *F*(5, 145) = 0.43, *p* = 0.83, *η^2^_p_* = 0.01.

## 4 Discussion

There have been discrepancies over whether readers could revise incorrect predictive inferences (e.g., Potts et al., 1988; Nahatame, 2014; Pérez et al., 2019; Xu et al., 2023). We tried to delve into the revision mechanisms within a longer period with more information coming in to support the alternative inferences consistent with new information inputs. By doing so, we aimed to trace down the time course of predictive inference revision and the amount of information needed to fulfill such revision to provide evidence for the incremental nature of the revision procedure. We expected to discover successful revision of the initially strongly activated predictive inferences at a later stage in reading comprehension after encountering conflicting information. We also hoped to find evidence for the lingering disruption effects of the disconfirmed predictive inferences even after readers have achieved successful revision.

Our results showed reduced mean amplitude data in ROIs associated with P3a for Target 2 compared with those of Target 1. The amplitude reduction shows that readers could detect the mismatch between the Target 1 representing the primary predictive inferences and the following information supporting the alternative predictive inferences after receiving conflicting information with the primarily strongly activated inferences. There was also a reduction in P3b mean amplitude data for Target 2, indicating that readers could further activate the alternative predictive inferences and disconfirm the now-inconsistent predictive inferences. Moreover, the reduction in mean amplitude data in ROIs associated with N400 when reading Target 2 suggests that readers could integrate the alternative predictive inferences in time. Furthermore, smaller mean amplitude data of P600 when reading Target 2 shows that there are processing costs for the disconfirmed predictive inferences and readers are making efforts in the second-pass re-analysis or prolonged attempts to make sense of the input. Reduction in the mean amplitude data of the f-PNP component when reading Target 2 suggests that readers could suppress or revise the disconfirmed predictive inferences when more information comes in to support the once-weakly predictive inference alternatives. Integration, reanalysis, and revision procedure could only be detected at a later period with sufficient amount of information supportive of the alternative predictive inferences. In addition, these results also reflect the influences of the disconfirmed primary predictive inferences. Though having been disconfirmed by the primary predictive inferences, the preliminary predictive inferences could still struggle with the alternative ones and exert disruptions on the following reading comprehension process.

Our findings are in line with those previous studies which discovered successful revision of predictive inferences (e.g., Nahatame, 2014; Pérez et al., 2015, 2016, 2019). Yet our findings are different from those in other studies showing immediate integration with the alternative predictive inferences and revision of the disconfirmed primary predictive inferences after readers have encountered information conflicting with the primary ones (e.g., Pérez et al., 2015, 2019). The current study adapted the experimental paradigm to that of Pérez et al. (2015) by inspecting the whole predictive inference revision procedure at a later time than that of the previous study. We contrasted ERP components of the two Target words at the very end of the last sentence of each short passage instead of examining the differences at the last but one sentence of three different versions. More importantly, we took into consideration more ERP components and analyzed a wider range of ERP components including P3a, P3b, N400, P600 and f-PNP, with the later two being more popular as indices of prediction revision. P3b in our current study was taken as an index of disconfirming inconsistent predictive inferences instead of an index of inference revision as in Pérez et al. (2015). As a result, we only detected integration with the alternative inferences and revision of the primary inferences at a later stage of the whole processing procedure.

### 4.1 Mismatch detection

Reduction in P3a mean amplitude data when reading Target 2 suggests that the word representing the once weakly activated predictive inference alternative is more consistent with the current situation model than the primarily strongly activated but later revised predictive inference. Readers are keen on detecting the mismatching information during reading comprehension. This is in accordance with the two-component model of evaluative comprehension (Isberner and Richter, 2014) that the initial detection of inconsistencies is a routine part of comprehension. And for the RI-Val model, the detection processes are memory-based and carried out routinely and efficiently, requiring few demands on cognitive resources (Richter et al., 2009). Therefore, the detection of mismatch is almost automatic.

### 4.2 Inference disconfirmation

The significant reduction in P3b mean amplitude data for Target 2 than Target 1 shows that readers could suppress or disconfirm inconsistent predictive inferences when mismatch information follows (e.g., Iseki, 2006; Pérez et al., 2015). According to the Structure Building Framework (Gernsbacher, 1990; Gernsbacher, 1997), activation levels of the activated memory nodes are subject to the mechanisms of suppression. Through suppression, inappropriate information is made unavailable while with enhancement, appropriate information becomes more easily available (Gernsbacher, 1997). The enhancement in P3b mean amplitude data of Target 1 showed readers’ efforts to update the once-activated but no-longer-relevant information, or in other words, the suppression procedure.

### 4.3 Delayed alternative inference integration

The significant differences of N400 mean amplitude data for Target 1 and Target 2 suggest easier integration of the latter, which represents the alternative predictive inferences. This result is consistent with findings of Pérez et al. (2015, 2019) which found more negativity in the revise condition than the no revise and neutral conditions for participants with high working memory capacity. These findings indicate that readers could integrate the alternative predictive inferences upon encountering the inconsistent information input. Yet, the results are different from those of Xu et al. (2023), which failed to find either successful integration of the alternative predictive inferences while reading the conflicting sentences. Major differences between the present experiment and others lie in the specific times at which integration was examined. By contrasting the results in the current study and those of previous research, we infer that the alternative inferences could be integrated within readers’ situation model, but with a delay.

### 4.4 Disconfirmed inference costs

There was a significant difference for P600 data in the two target word conditions, showing costs of disconfirming the originally dominant predictive inferences. Previous studies showed that syntactic reanalysis in “garden-path sentences” happened immediately when comprehenders encountered information in conflicts with their initial analysis (Frazier and Rayner, 1982). The reduction in the amplitude data of P600 for Target 2 in our experiment suggests that the costs of readers in their semantic/syntactic adaptation or updating when incoming words are not explained away by the predictions that readers have primarily made. This suggests that the disconfirmed predictive inferences remain to be active in readers’ memory representation though disconfirmed by conflicting information input, incurring cognitive processing costs. It is possible that the already-encoded primary predictive inferences still lingers on in readers’ working memory representation, although their activation levels have been reduced after the primary inferences have been disconfirmed.

### 4.5 Delayed inference revision

The reduction in the mean amplitude data of f-PNP showed that readers had successfully revised the disconfirmed predictive inferences after encountering conflicting information with the primary inferences. Unlike previous investigation into predictive inference revision, which found immediate revision of inconsistent inferences upon accessing conflicting information inputs (e.g., Pérez et al., 2015, 2019), the current study detected the successful revision procedure, even with a delay. Studies of prediction at the sentence level have found that in natural sentence processing, predictions are instantaneously updated whenever new information becomes available (e.g., Szewczyk, 2018). However, information that disconfirms a strong prediction may have a disruptive effect on processing and may prevent comprehenders from immediately revising their predictions (Federmeier et al., 2007; van Petten and Luka, 2012).

In sum, results of the current study corresponds to those of other studies (e.g., Nahatame, 2014; Pérez et al., 2015), which shows that readers could detect the mismatch, disconfirm the inconsistent predictive inferences, integrate the predictive inference alternatives, and revise the primarily highly activated predictive inferences. The results of our experiment suggest that readers could successfully integrate an initially weakly activated alternative predictive inference after receiving enough new supporting information. This might also indicate that the very strong initial contextual constraint could hardly be disconfirmed by insufficient local disconfirmation. In addition, the primarily generated but later disconfirmed predictive inferences still exert influences at later reading comprehension stages This is in accordance with such theories as the KReC framework (Kendeou and O’Brien, 2014) holding that even though the revised information has lost activation and becomes less accessible for the comprehension of subsequent texts, it still exists in the long-term memory representation, and it can still be reactivated and disrupt comprehension. This could explain why there is delayed revision of the disconfirmed predictive inferences.

With all these indications, it is possible that the revised predictive inference has really been deactivated and the activation levels of the originally low-competitive alternative inferences have been enhanced with more supporting information. This might well explain the failure of integration or revision in some studies. For example, Xu et al. (2023) found that readers failed to integrate the low-competitive alternative predictive inferences into their long-term representation in Experiment 1 in which little attention was paid to the activation levels of an alternative predictive inference in the first place. Experiment 2 managed to ensure a high contextual constraint for the preliminary predictive inference while providing low probability of eliciting other strongly constrained alternative predictive inferences, thus eliciting only weakly constrained alternatives that might be derived from the introduction at the same time. Integration of the alternative predictive inferences, though supported by the disconfirming information, failed to happen immediately after the disconfirmation of the conflicting information. By using the P600 component as an index of predictive inference re-analysis or revision procedure in the experiments, the research did not find evidence of successful revision by readers upon the disconfirming information, either. Failure to integrate the alternative predictive differences or revise the disconfirmed predictive inferences immediately upon encountering conflicting new inputs can happen for many reasons.

There are three possible explanations for the delayed integration of the alternative predictive inferences and revision of the disconfirmed predictive inferences. Firstly, for materials used in Xu et al. (2023), the activation of a newly revised predictive inference came mainly from a single sentence containing a contradictory context. There was a lack of enough resonance of the alternative predictive inferences from the contextual information and the disconfirming information in the fourth sentence. Therefore, it might be contextually insufficient for the activation and encoding of the alternative predictive inferences. The alternative inferences that were initially weakly activated were not probable enough to affect the processing of the target inference (see Cranford and Moss, 2023). According to the minimal encoding hypothesis (McKoon and Ratcliff, 1992), when there is another possible consequence, only some of the semantic features of the inferred proposition are encoded into readers’ working memory. With more supporting information, however, one of the alternatives can become more specific and highly activated. Secondly, the activation levels of an alternative predictive inference can be enhanced with more supporting information that comes in during reading comprehension. According to findings in previous studies, predictive inferences are activated in a general way and are modified or become quickly lost if not supported by the following information (Keefe and McDaniel, 1993). Readers might initially draw general and flexible predictive inferences with more than one possibility. They try to make them more specific predictive inferences as the reading process goes on (McKoon and Ratcliff, 1986; Lassonde and O’Brien, 2009). Moreover, when the amount of information supporting the alternative predictive inferences increases, the activation of the once low-competitive predictive inferences will be enhanced. This assumption could be supported by Harmon (2005), in which the researcher adopted a probe-naming task to detect the activation of a primary predictive inference. The results indicated that the primary predictive inference could be activated if there was low distracting information before the inference-evoking sentence. It was concluded that with less distracting information, the probability of a predictive inference would be increased. Conversely, the more distracting information there was, the less probable the predictive inferences might happen. Successful integration detected at a later stage suggests that a little bit more supporting information could specify and further activate the alternative predictive inference to make it more relevant to the current situation model, thus easing its integration. Thirdly, the delayed integration or revision could also be viewed as a spill-over effect, in which readers do not begin resolving the inconsistency until after moving past the contradictory sentence. The delayed integration or revision might be related to a spill-over effect in contradiction solution. Previous studies constructed post-contradictory sentences to check the spill-over effects of predictive inference activation (e.g., Klin et al., 1999a, 1999b; Weingartner et al., 2003). Consistent with the findings of previous studies, the revision process is more complete after the contradiction appears and some integration processes have been shown to be delayed until the end of the sentence (Rayner et al., 1989; Guzmán and Klin, 2000). In other words, the delayed integration or revision aligns with the findings of previous studies that have found observed activation of predictive inferences with a longer delay. For instance, in Experiment 1 of Weingartner et al. (2003), the Inter-Stimulus Interval between the critical context and the naming probe was increased from the 500 ms in Klin et al. (1999a) to 1,500 ms. With this longer delay, evidence of predictive inferences was provided in the distractor version. Lastly, the revised information may still exists in the long-term memory representation of readers and exerts influences on subsequent reading processes. According to the KReC framework, this deactivated information can still be reactivated, even though it has lost activation and becomes less accessible during the comprehension of follow-up information. Prior studies have found that readers have difficulties removing the incorrect information from their working memory (e.g., Wilkes and Leatherbarrow, 1988; Johnson and Seifert, 1994; Nahatame, 2014). The outdated information might be reactivated whenever supported by incoming information (e.g., Campion, 2004; Guéraud et al., 2005). Similarly, Weingartner et al. (2003) discovered that the targeted inference was not deleted in the presence of alternative consequences.

In addition, the conflicts between global and local constraints could help further explain the results in our current study. For materials used in Xu et al. (2023), there were strong contextual constraints for eliciting the primary predictive inferences. However, its contextual support was not sufficient for the activation and encoding of the alternative predictive inferences, because predictive inferences generated by the global context (the introduction) conflicted with the local context when readers read containing contradictory information against the global context. According to the fruitful results concerning the interplay between global and local context in research areas such as the processing of counterfactual conditionals (e.g., Ferguson et al., 2008; Nieuwland and Martin, 2012), fictional contexts (e.g., Nieuwland and Van Berkum, 2006; Filik and Leuthold, 2008) and concessive sentences (e.g., Xiang and Kuperberg, 2015; Rich and Harris, 2022), the global context serves to constrain the effect of the local context on predictability, even at the earliest stages of lexical processing. Although the traditional view held that obtaining evidence for the activation of predictive inferences only occurred when the strong supportive context immediately preceding the point at which activation was measured (e.g., McKoon and Ratcliff, 1986; Keefe and McDaniel, 1993), recent studies have proved that activation does not depend on the immediate local context and information from earlier portions of a text can play an important role in the overall pattern of activation (e.g., Cook et al., 2001; Guéraud et al., 2008). According to the incremental processing point of view, contextual support can come from anywhere in the text and global context can mediate expectations generated by local constraints (Lassonde and O’Brien, 2009; Rich and Harris, 2022). Facilitation for predictable words has been found in the presence of highly constraining local contexts (words) directly preceding the critical word (e.g., Fitzsimmons and Drieghe, 2013; Chow and Chen, 2020). Therefore, results of the current study evidence that when the global context is in conflicts with the local context, the global context constrains the effects of the local contexts on predictive inference making and revision.

## 5 Conclusion

In conclusion, the low-competitive alternative inferences received more activation with the supporting information from both the disconfiming information and the disambiguating information in the ERP sentences ended with Target 2 representing the alternative predictive inference. More sufficient information supporting the primarily weakly activated predictive inferences enables readers to achieve successful integration and revision at a later stage of reading comprehension. Our findings are supportive of the slow, incremental, and conservative nature of information revision as proposed by the KReC framework in that readers do not engage themselves in the immediate process of predictive inference revision upon receiving conflicting information during their reading comprehension. Instead, they hold the disconfirmed inferences till enough information comes in to enrich the global context and enhance the support for alternative predictive inferences before they successfully revise the inconsistent predictive inferences. Our results also add evidence to the proposals of the KReC framework in that the primarily strongly supported predictive inferences, even though disconfirmed, have been encoded into readers’ working memory and could hardly be deleted and could be reactivated whenever they are supported by sufficient amount of new information. In view of the conservative nature of predictive inference revision mechanism, future studies should trace further to find out whether the inconsistent, but primarily more strongly generated predictive inferences would be deleted and totally replaced by the alternative predictive inferences. Similarly, whether predictive inference revision happens when the alternative predictive inferences are of almost equal activation levels to the primary predictive inferences is also worthy of further investigation. In addition, the effects of the global and local contexts on predictive inference revision still awaits more investigation.

## Data Availability

The datasets presented in this study can be found in online repositories. The names of the repository/repositories and accession number(s) can be found below: https://osf.io/qdv9a/.

## References

[ref1] Aurnhammer C. Delogu F. Schulz M. Brouwer H. Crocker M. W. (2021). Retrieval (N400) and integration (P600) in expectation-based comprehension. PLoS One 16:e0257430. doi: 10.1371/journal.pone.0257430, PMID: 34582472 PMC8478172

[ref2] Bar M. (2011). Predictions in the brain: using our past to generate a future. Oxford: Oxford University Press.

[ref3] Boudewyn M. A. (2015). Individual differences in language processing: electrophysiological approaches. Lang. Linguist. Compas. 9, 406–419. doi: 10.1111/lnc3.12167

[ref4] Brothers T. Wlotko E. W. Warnke L. Kuperberg G. R. (2020). Going the extra mile: effects of discourse context on two late positivities during language comprehension. Neurobiol. Lang. 1, 135–160. doi: 10.1162/nol_a_00006, PMID: 32582884 PMC7313229

[ref5] Cai Q. Brysbaert M. (2010). SUBTLEX-CH: Chinese word and character frequencies based on film subtitles. PLoS One 5:e10729. doi: 10.1371/journal.pone.0010729, PMID: 20532192 PMC2880003

[ref6] Calvo M. G. Castillo M. D. (1996). Predictive inferences occur on-line, but with delay: convergence of naming and reading times. Discl. Process. 22, 57–78. doi: 10.1080/01638539609544966

[ref7] Campion N. (2004). Predictive inferences are represented as hypothetical facts. J. Mem. Lang. 50, 149–164. doi: 10.1016/j.jml.2003.10.002

[ref8] Ceháková M. Chromý J. (2023). Garde-path sentences and the diversity of their (mis)representations. PLoS One 18:e0288817. doi: 10.1371/journal.pone.0288817, PMID: 37463143 PMC10353815

[ref9] Chow W. Chen D. (2020). Predicting (in)correctly: listeners rapidly use unexpected information to revise their predictions. J. Cogn. Neurosci. 35, 1149–1161. doi: 10.1080/23273798.2020.1733627

[ref10] Clark A. (2013). Whatever next? Predictive brains, situated agents, and the future of cognitive science. Behav. Brain Sci. 36, 181–204. doi: 10.1017/S0140525X12000477, PMID: 23663408

[ref11] Cook A. E. Limber J. E. O’Brien E. J. (2001). Situation-based context and the availability of predictive inferences. J. Mem. Lang. 44, 220–234. doi: 10.1006/jmla.2000.2744

[ref9001] Cranford E. A. Moss J. (2019). Generating predictive inferences when multiple alternatives are available. Discl. Process. 56, 289–309. doi: 10.1080/0163853x.2018.1497921

[ref12] Cranford E. A. Moss J. (2023). Representation of predictive inferences when multiple alternatives are available. Discl. Process. 60, 181–201. doi: 10.1080/0163853X.2023.2196915

[ref13] Federmeier K. D. Kutas M. Schul R. (2010). Age-related and individual differences in the use of prediction during language comprehension. Brain Lang. 115, 149–161. doi: 10.1016/j.bandl.2010.07.006, PMID: 20728207 PMC2975864

[ref14] Federmeier K. D. Wlotko E. W. De Ochoa-Dewald E. Kutas M. (2007). Multiple effects of sentential constraint on word processing. Brain Res. 1146, 75–84. doi: 10.1016/j.brainres.2006.06.101, PMID: 16901469 PMC2704150

[ref15] Ferguson H. J. Sanford A. J. Leuthold H. (2008). Eye-movements and ERPs reveal the time course of processing negation and remitting counterfactual worlds. Brain Res. 1236, 113–125. doi: 10.1016/j.brainres.2008.07.099, PMID: 18722356

[ref16] Filik R. Leuthold H. (2008). Processing local pragmatic anomalies in fictional contexts: evidence from the N400. Psychophysiology 45, 554–558. doi: 10.1111/j.1469-8986.2008.00656.x, PMID: 18282200

[ref18] Fitzsimmons G. Drieghe D. (2013). How fast can predictability influence word skipping during reading? J. Exp. Psychol.-Learn. Mem. Cogn. 39, 1054–1063. doi: 10.1037/a0030909, PMID: 23244054

[ref19] Frazier L. Rayner K. (1982). Making and correcting errors during sentence comprehension: eye movements in the analysis of structurally ambiguous sentences. Cogn. Psychol. 14, 178–210. doi: 10.1016/0010-0285(82)90008-1

[ref20] Friedman D. Cycowicz Y. M. Gaeta H. (2001). The novelty P3: an event-related brain potential (ERP) sign of the brain’s evaluation of novelty. Neurosci. Biobehav. Rev. 25, 355–373. doi: 10.1016/s0149-7634(01)00019-7, PMID: 11445140

[ref9003] Gernsbacher M. A. (1990). Language comprehension as structure building. Hillsdale, NJ: Erlbaum.

[ref21] Gernsbacher M. A. (1997). Two decades of structure building. Discourse Process. 23, 265–304. doi: 10.1080/01638539709544994, PMID: 25484476 PMC4255941

[ref9002] Gong Y. (1983). Manual for WAIS-RC. Hunan: Hunan Medical University.

[ref22] Guéraud S. Harmon M. E. Peracchi K. A. (2005). Updating situation models: the memory-based contribution. Discl. Process 39, 243–263. doi: 10.1207/s15326950dp3902&3_8

[ref23] Guéraud S. Tapiero I. O’Brien E. J. (2008). Context and the activation of predictive inferences. Psychon. Bull. Rev. 15, 351–356. doi: 10.3758/PBR.15.2.351 18488651

[ref24] Guzmán A. E. Klin C. M. (2000). Maintaining global coherence in reading: the role of sentence boundaries. Mem. Cogn. 28, 722–730. doi: 10.3758/bf03198406, PMID: 10983445

[ref25] Hagoort P. Hald L. A. Bastiaansen M. C. M. Petersson K. M. (2004). Integration of word meaning and world knowledge in language comprehension. Science 304, 438–441. doi: 10.1126/science.1095455, PMID: 15031438

[ref26] Harmon M. E. (2005). Factors affecting the activation of predictive inferences [Unpublished doctoral dissertation]. Durham: University of New Hampshire.

[ref27] Hodapp A. Rabovsky M. (2021). The N400 ERP component reflects an error-based implicit learning signal during language comprehension. Eur. J. Neurosci. 54, 7125–7140. doi: 10.1111/ejn.15462, PMID: 34535935

[ref28] Isberner M. B. Richter T. (2014). “Comprehension and validation: separable stages of information processing? A case for epistemic monitoring in language” in Processing inaccurate information. eds. Rapp D. N. Braasch J. L. G. (Cambridge, MA: MIT Press), 245–276.

[ref29] Iseki R. (2006). Text rikai ni okeru on-line suiron seisei no kiteiin: Seigousei to access kanousei no hikaku [which regulates on-line inference generation in text comprehension, coherence or accessibility?]. Cogn. Stud. 13, 205–224.

[ref30] Johnson H. M. Seifert C. M. (1994). Sources of the continued influence effect: when misinformation in memory affects later inferences. J. Exp. Psychol.-Learn. Mem. Cogn. 20, 1420–1436. doi: 10.1037/0278-7393.20.6.1420

[ref31] Kamide Y. Altmann G. T. M. Haywood S. L. (2003). The time-course of prediction in incremental sentence processing: evidence from anticipatory eye movements. J. Mem. Lang. 49, 133–156. doi: 10.1016/S0749-596X(03)00023-8

[ref32] Keefe D. E. McDaniel M. A. (1993). The time course and durability of predictive inferences. J. Mem. Lang. 32, 446–463. doi: 10.1006/jmla.1993.1024

[ref33] Kendeou P. O’Brien E. J. (2014). “The knowledge revision components (KReC) framework: processes and mechanisms” in Processing inaccurate information: theoretical and applied perspectives from cognitive science and the educational sciences. eds. Rapp D. N. Braasch J. L. G. (Cambridge, MA: MIT Press), 353–377.

[ref34] Klin C. M. Guzmán A. E. Levine W. H. (1999a). Prevalence and persistence of predictive inferences. J. Mem. Lang. 40, 593–604. doi: 10.1006/jmla.1998.2628

[ref35] Klin C. M. Murray J. D. Levine W. H. Guzmán A. E. (1999b). Forward inferences: from activation to long-term memory. Discl. Process. 27, 241–260. doi: 10.1080/01638539909545062

[ref36] Kok A. (2001). On the utility of P3 amplitude as a measure of processing capacity. Psychophysiology 38, 557–577. doi: 10.1017/S0048577201990559, PMID: 11352145

[ref38] Kuperberg G. R. (2013). “The proactive comprehender: what event-related potentials tell us about the dynamics of reading comprehension” in Unraveling the behavioral, neurobiological, and genetic components of reading comprehension. eds. Miller B. Cutting L. McCardle P. (Baltimore: Paul Brookes Publishing), 176–192.

[ref39] Kuperberg G. R. Brothers T. Wlotko E. W. (2020). A tale of two positivities and the N400: distinct neural signatures are evoked by confirmed and violated predictions at different levels of representation. J. Cogn. Neurosci. 32, 12–35. doi: 10.1162/jocn_a_01465, PMID: 31479347 PMC7299186

[ref40] Kuperberg G. Caplan D. Sitnikova T. Eddy M. Holcomb P. (2006). Neural correlates of processing syntactic, semantic, and thematic relationships in sentences. Lang. Cogn. Process. 21, 489–530. doi: 10.1080/01690960500094279

[ref41] Kuperberg G. R. Jaeger T. F. (2016). What do we mean by prediction in language comprehension? Lang. Cogn. Neurosci. 31, 32–59. doi: 10.1080/23273798.2015.1102299, PMID: 27135040 PMC4850025

[ref42] Kutas M. (1993). In the company of other words: electrophysiological evidence for single-word and sentence context effects. Lang. Cogn. Process. 8, 533–572. doi: 10.1080/01690969308407587

[ref43] Kutas M. Federmeier K. D. (2011). Thirty years and counting: finding meaning in the N400 component of the event related brain potential (ERP). Annu. Rev. Psychol. 62, 621–647. doi: 10.1146/annurev.psych.093008.131123, PMID: 20809790 PMC4052444

[ref44] Lassonde K. A. O’Brien E. J. (2009). Contextual specificity in the activation of predictive inferences. Discl. Process. 46, 426–438. doi: 10.1080/01638530902959620

[ref45] Linden D. E. J. (2005). The P300: where in the brain is it produced and what does it tell us? Neuroscientist 11, 563–576. doi: 10.1177/1073858405280524 16282597

[ref46] McKoon G. Ratcliff R. (1986). Inferences about predictable events. J. Exp. Psychol.-Learn. Mem. Cogn. 12, 82–91. doi: 10.1037/0278-7393.12.1.82 2949049

[ref47] McKoon G. Ratcliff R. (1992). Inference during reading. Psychol. Rev. 99, 440–466. doi: 10.1037/0033-295X.99.3.440 1502273

[ref48] Nahatame S. (2014). Making and revising predictive inferences in Japanese EFL learners’ reading comprehension. Unpublished doctoral dissertation. University of Tsukuba.

[ref49] Nieuwland M. S. Barr D. J. Bartolozzi F. Busch-Moreno S. Darley E. Donaldson D. I.. (2020). Dissociable effects of prediction and integration during language comprehension: evidence from a large-scale study using brain potentials. Philos. Trans. R. Soc. Lond. 375:20180522. doi: 10.1098/rstb.2018.0522, PMID: 31840593 PMC6939355

[ref50] Nieuwland M. S. Martin A. E. (2012). If the real world were irrelevant, so to speak: the role of propositional truth value in counterfactual sentence comprehension. Cognition 122, 102–109. doi: 10.1016/j.cognition.2011.09.001, PMID: 21962826

[ref51] Nieuwland M. S. Van Berkum J. J. A. (2006). When peanuts fall in love: N400 evidence for the power of discourse. J. Cogn. Neurosci. 18, 1098–1111. doi: 10.1162/jocn.2006.18.7.1098, PMID: 16839284

[ref52] Oldfield R. C. (1971). The assessment and analysis of handedness: the Edinburgh inventory. Neuropsychologia 9, 97–113. doi: 10.1016/0028-3932(71)90067-4 5146491

[ref54] Pérez A. Cain K. Castellanos M. C. Bajo T. (2015). Inferential revision in narrative texts: an ERP study. Mem. Cogn. 43, 1105–1135. doi: 10.3758/s13421-015-0528-0, PMID: 26047776

[ref55] Pérez A. Hansen L. Bajo T. (2019). The nature of first and second language processing: the role of cognitive control and L2 proficiency during text-level comprehension. Biling.-Lang. Cogn. 22, 930–948. doi: 10.1017/S1366728918000846

[ref56] Pérez A. Joseph H. S. Bajo T. Nation K. (2016). Evaluation and revision of inferential comprehension in narrative texts: an eye movement study. Lang. Cogn. Process. 31, 549–566. doi: 10.1080/23273798.2015.1115883

[ref57] Pérez A. Schmidt E. Kourtzi Z. Tsimpli I. (2020). Multimodal semantic revision during inferential processing: the role of inhibitory control in text and picture comprehension. Neuropsychologia 138:107313. doi: 10.1016/j.neuropsychologia.2019.107313, PMID: 31904356

[ref58] Polich J. (2003). “Theoretical overview of P3a and P3b” in Detection of change. ed. Polich J. (Dordrecht: Kluwer Academic), 83–98.

[ref59] Polich J. (2007). Updating P300: an integrative theory of P3a and P3b. Clin. Neurophysiol. 118, 2128–2148. doi: 10.1016/j.clinph.2007.04.019, PMID: 17573239 PMC2715154

[ref60] Potts G. R. Keenan J. M. Golding J. M. (1988). Assessing the occurrence of elaborative inference: lexical decision versus naming. J. Mem. Lang. 27, 399–415. doi: 10.1016/0749-596X(88)90064-2

[ref61] Rapp D. N. Kendeou P. (2007). Revising what readers know: updating text representations during narrative comprehension. Mem. Cogn. 35, 2019–2032. doi: 10.3758/BF03192934, PMID: 18265617

[ref62] Rasenberg M. Rommers J. van Bergen G. (2020). Anticipating predictability: an ERP investigation of expectation-managing discourse markers in dialogue comprehension. Lang. Cogn. Process. 35, 1–16. doi: 10.1080/23273798.2019.1624789

[ref63] Rayner K. Sereno S. C. Morris R. K. Schmauder A. R. Clifton C. (1989). Eye movements and on-line language comprehension processes. Lang. Cogn. Process. 4, SI21–SI49. doi: 10.1080/01690968908406362

[ref64] Rich S. Harris J. A. (2022). Global expectations mediate local constraint: evidence from concessive structures. Lang. Cogn. Neurosci. 38, 302–327. doi: 10.1080/23273798.2022.2114598

[ref65] Richter T. Schroeder S. Wöhrmann B. (2009). You don’t have to believe everything you read: background knowledge permits fast and efficient validation of information. J. Pers. Soc. Psychol. 96, 538–558. doi: 10.1037/a0014038, PMID: 19254102

[ref66] Schneider W. Eschman A. Zuccolotto A. (2002). E-prime user’s guide. Pittsburgh: Psychology Software Tools Inc.

[ref67] Szewczyk J. M. (2018) Prediction-inconsistent information leads to prediction updating – an ERP study on sentence comprehension. Paper presentation, The 31st Annual CUNY sentence processing conference, Davis, CA, USA

[ref68] Tanenhaus M. K. Spivey-Knowlton M. J. Eberhard K. M. Sedivy J. C. (1995). Integration of visual and linguistic information in spoken language comprehension. Science 268, 1632–1634. doi: 10.1126/science.7777863 7777863

[ref70] van den Broek P. Beker K. Oudega M. (2015). “Inference generation in text comprehension: automatic and strategic processes in the construction of a mental representation” in Inferences during reading. eds. O’Brien E. J. Cook A. E. Lorch R. F. (Cambridge: Cambridge University Press), 94–121.

[ref71] Van Petten C. Luka B. J. (2012). Prediction during language comprehension: benefits, costs, and ERP components. Int. J. Psychophysiol. 83, 176–190. doi: 10.1016/j.ijpsycho.2011.09.015, PMID: 22019481

[ref72] Weingartner K. M. Guzmán A. E. Levine W. H. Klin C. M. (2003). When throwing a vase has multiple consequences: minimal encoding of predictive inferences. Discl. Process. 36, 131–146. doi: 10.1207/S15326950DP3602_3

[ref73] Wilkes A. L. Leatherbarrow M. (1988). Editing episodic memory following the identification of error. Q. J. Exp. Psychol. Sect A-Hum. Exp. Psychol. 40, 361–387. doi: 10.1080/02724988843000168

[ref74] Wright H. H. Newhoff M. (2002). Age-related differences in inference revision processing. Brain Lang. 80, 226–239. doi: 10.1006/brln.2001.2595, PMID: 11827445

[ref75] Xiang M. Kuperberg G. (2015). Reversing expectations during discourse comprehension. Lang. Cogn. Neurosci. 30, 648–672. doi: 10.1080/23273798.2014.995679, PMID: 25914891 PMC4405243

[ref76] Xu F. Fan L. Tian L. Cheng L. (2023). Making and revising predictive inferences during Chinese narrative text reading: evidence from an electrophysiological study. Front. Psychol. 13:1061725. doi: 10.3389/fpsyg.2022.1061725, PMID: 36710800 PMC9880981

[ref77] Zirnstein M. van Hell J. G. Kroll J. F. (2018). Cognitive control ability mediates prediction costs in monolinguals and bilinguals. Cognition 176, 87–106. doi: 10.1016/j.cognition.2018.03.001, PMID: 29549762 PMC5953823

